# Evolutionary Analysis of Severe Acute Respiratory Syndrome Coronavirus 2 (SARS-CoV-2) Reveals Genomic Divergence with Implications for Universal Vaccine Efficacy

**DOI:** 10.3390/vaccines8040591

**Published:** 2020-10-08

**Authors:** Nanda Kumar Yellapu, Shachi Patel, Bo Zhang, Richard Meier, Lisa Neums, Dong Pei, Qing Xia, Duncan Rotich, Rosalyn C. Zimmermann, Emily Nissen, Shelby Bell-Glenn, Whitney Shae, Jinxiang Hu, Prabhakar Chalise, Lynn Chollet-Hinton, Devin C. Koestler, Jeffery A. Thompson

**Affiliations:** 1 Department of Biostatistics & Data Science, University of Kansas Medical Center, 3901 Rainbow Boulevard, Kansas City, KS 66160, USA; nyellapu@kumc.edu (N.K.Y.); spatel14@kumc.edu (S.P.); b021z055@kumc.edu (B.Z.); rmeier2@kumc.edu (R.M.); lneums@kumc.edu (L.N.); dpei@kumc.edu (D.P.); q508x072@kumc.edu (Q.X.); duncancheru@gmail.com (D.R.); e617n596@kumc.edu (E.N.); sbell5@kumc.edu (S.B.-G.); wwhite7@kumc.edu (W.S.); jhu2@kumc.edu (J.H.); pchalise@kumc.edu (P.C.); lhinton@kumc.edu (L.C.-H.); 2 Department of Cancer Biology, University of Kansas Medical Center, 3901 Rainbow Boulevard, Kansas City, KS 66160, USA; rzimmermann@kumc.edu

**Keywords:** COVID-19, SARS-CoV-2, phylogenetic analysis, genomic divergence, vaccine development

## Abstract

Coronavirus disease (COVID-19), caused by severe acute respiratory syndrome coronavirus 2 (SARS-CoV-2), is one of the pressing contemporary public health challenges. Investigations into the genomic structure of SARS-CoV-2 may inform ongoing vaccine development efforts and/or provide insights into vaccine efficacy to fight against COVID-19. Evolutionary analysis of 540 genomes spanning 20 different countries/territories was conducted and revealed an increase in the genomic divergence across successive generations. The ancestor of the phylogeny was found to be the isolate from the 2019/2020 Wuhan outbreak. Its transmission was outlined across 20 countries/territories as per genomic similarity. Our results demonstrate faster evolving variations in the genomic structure of SARS-CoV-2 when compared to the isolates from early stages of the pandemic. Genomic alterations were predominantly located and mapped onto the reported vaccine candidates of structural genes, which are the main targets for vaccine candidates. S protein showed 34, N protein 25, E protein 2, and M protein 3 amino acid variations in 246 genomes among 540. Among identified mutations, 23 in S protein, 1 in E, 2 from M, and 7 from N protein were mapped with the reported vaccine candidates explaining the possible implications on universal vaccines. Hence, potential target regions for vaccines would be ideally chosen from the structural regions of the genome that lack high variation. The increasing variations in the genome of SARS-CoV-2 together with our observations in structural genes have important implications for the efficacy of a successful universal vaccine against SARS-CoV-2.

## 1. Introduction

Severe acute respiratory syndrome coronavirus 2 (SARS-CoV-2) belongs to the viral family *Coronaviridae*, which includes a large number of viruses found in mammals and birds. The first human coronavirus was identified in the 1960s after an outbreak caused a large number of respiratory infections [1,2]. The number of scientific contributions related to SARS-CoV-2 has increased exponentially after its emergence in southern China in December 2019. Epidemiological investigations and genome sequencing experiments revealed SARS-CoV-2 as the etiological agent of the COVID-19 pandemic [3]. Prior to the emergence of SARS-CoV-2, six other human coronaviruses have been reported: α-coronaviruses 229E and NL63, β-coronaviruses OC43 and HKU1, Middle East respiratory syndrome coronavirus (MERS-CoV), and SARS-associated coronavirus (SARS-CoV). Among those six, SARS-CoV and MERS-CoV have been shown to be associated with severe respiratory disease, high rates of acute lung injury, and correspondingly high mortality rates [4,5,6]. SARS-CoV-2 is divergent from all known human coronaviruses, including the 2012 MERS-CoV, which is believed to have originated from Saudi Arabia [7] and is associated with a rapid rate of transmission from human to human. Though both fall under the Betacoronavirus lineage-c, analysis of genome sequence, size, and organization reveals that MERS-CoV significantly differs from SARS-CoV [8,9].

The first infection of SARS-CoV-2 was reported from the city of Wuhan in Hubei province of China [10,11]. This led to the COVID-19 outbreak, which began in late December 2019 and continues to this day [12]. As of August 2020, more than 19 million confirmed cases of SARS-CoV-2 and 730,000 deaths have been reported. The global emergence and exponential increase of SARS-CoV-2 cases demands rapid scientific contributions to control and mitigate its impact.

**Figure 1:**
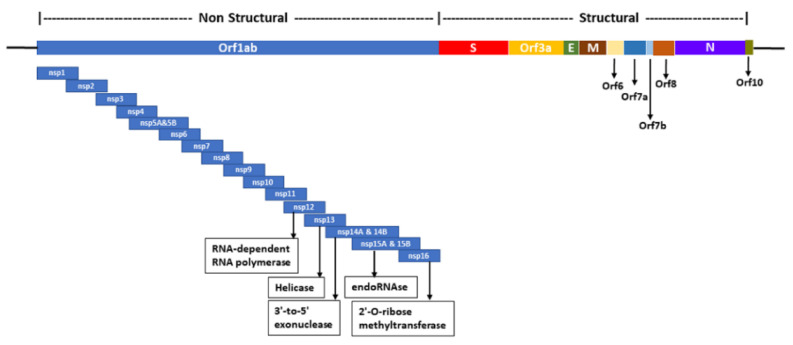
Genomic structure of SARS-CoV-2. The entire genome is classified as nonstructural and structural regions that produce multiple open reading frames (ORFs).

**Figure 2:**
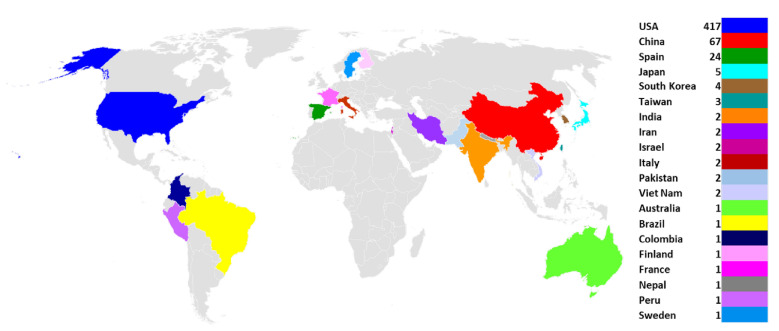
Distribution of SARS-CoV-2 genomes. A total of 540 genomes from 20 different countries are represented with 20 different color patterns.

The full genomic sequence of SARS-CoV-2 and its structural organization were first reported by the Yongzhen Zhang team in China [13]. The genome is arranged as 5′-untranslated region (UTR)-replicase complex (orf1ab)-structural proteins (Spike(S)-Envelope(E)-Membrane(M)-Nucleocapsid(N)) - 3′-UTR (Figure 1). The sequence was deposited in the National Center for Biotechnology Information (NCBI) GenBank in January 2020 and the annotation was subsequently provided (NC_045512) [14]. A total of 540 complete genome sequences from 20 different countries/territories with annotated information from NCBI were analyzed in the current study to provide plausible insights into the genomic divergence of SARS-CoV-2 (Figure 2). The pressing public health issues associated with this illness motivated us to conduct an evolutionary analysis of SARS-CoV-2 across different countries/territories, starting from the reference genome reported from China. The evolutionary analysis described herein helps to shed light on the genomic changes across both time and space (e.g., different countries/territories), as well as expanding our understanding of how genomic variations of SARS-CoV-2 may relate to the development/efficacy of vaccines and therapeutics.

To date, there have been several studies that have conducted phylogenetic analyses of SARS-CoV-2 based on a varying number of genomic sequences [15,16,17,18,19,20,21,22,23]. Phylogenetic analysis of 160 SARS-CoV-2 genomes revealed three central variants distinguished by random amino acid variations [24]. A four-genome phylogeny from Chile revealed two different viral variants coming from Europe and Asia [25]. The characterization and phylogenetic analysis of the first three genomes from Italy revealed a single amino acid variation in the surface glycoprotein [26]. A report from Europe on the phylogenetic analysis of two SARS-CoV-2 genomes revealed the introduction of novel variants describing the initial stages of viral evolution [27]. The Nextstrain database is continuously updating the information on the phylogenetic analysis and genomic divergence of SARS-CoV-2 with concomitant updates on the evolutionary changes [28]. Phylogenetic frameworks are essential tools to identify virus lineages that contribute to their active spread. Rambaut et al. proposed a rational and dynamic virus nomenclature for naming the expanding phylogenetic diversity of SARS-CoV-2 [29]. Their method was made tractable by constraining the number and depth of hierarchical lineage labels and focusing on active virus lineages that are spreading to wider locations. This nomenclature will assist in tracking and understanding the patterns and determinants of the global spread of SARS-CoV-2. In addition to the phylogenetics and divergence, in our current study we aimed to derive the impact of the observed evolutionary changes on the protein functionality and therapeutic interventions. The evolutionary analysis of 540 genomes from 20 different countries/territories described here allowed us to detect a greater number of variations, and correspondingly, the potential impact of such variations on the protein sequences and their implications on vaccine development.

## 2. Materials and Methods

### 2.1. Genome Sequence Data

Aiming to derive the genomic divergence of SARS-CoV-2 across different countries/territories, 540 complete genome sequences with annotated information were retrieved from NCBI—Virus datahub (https://www.ncbi.nlm.nih.gov/labs/virus/vssi/#/). The NCBI refseqs are supplied through three distinct pipelines (computed annotation, Entrez genomes, and LocusLink supported pipelines) curated on an ongoing basis [30]. The NCBI viral genome resource curates the submitted viral genome sequences with forced recalibration of the data to better provide extant sequence representations with enhanced reference information. This, in turn, increases the emphasis on leveraging the genome sequence data [31]. Such validated SARS-CoV-2 genome sequences were retrieved in FASTA format as available on 12 April 2020, and the sequence information is provided as supporting information (Files S1 & S2).

### 2.2. Phylogenetic Network Analysis

**Figure 3:**
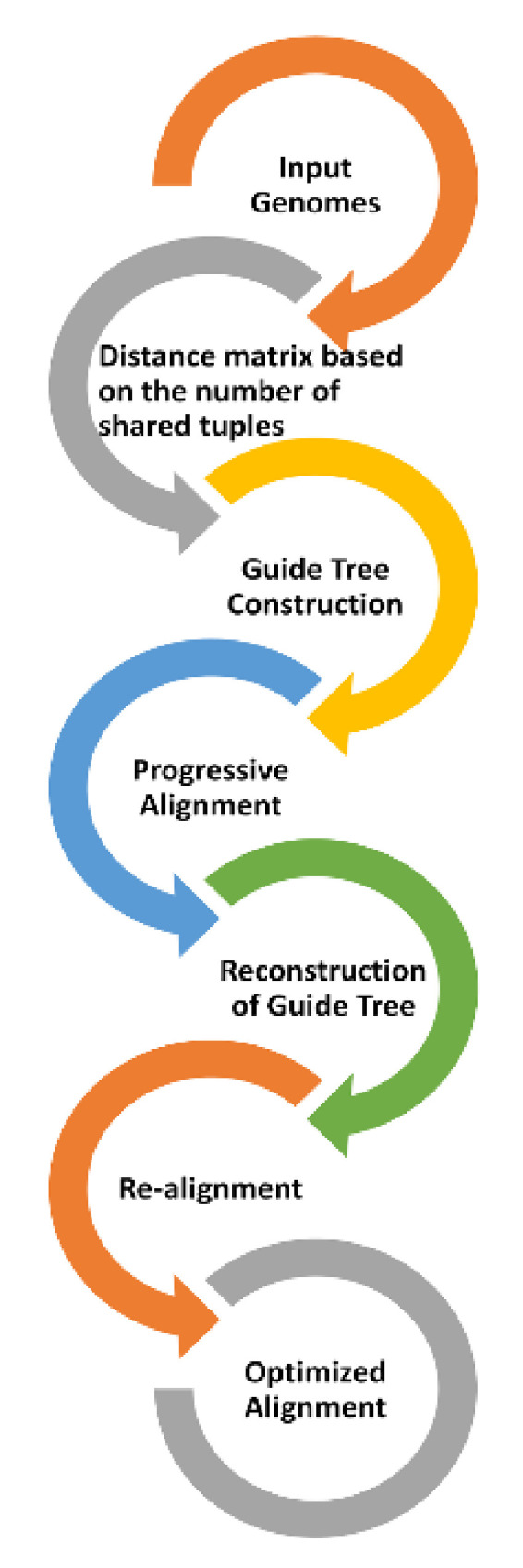
Multiple sequence alignment process by MAFFT v7.4.2. Multiple sequence alignment was carried out in a multistage process to generate the final accurate alignment. Subsequent phylogenetic analysis was conducted using MEGA-X.

A full-genomic sequence alignment was performed for the 540 sequences using MAFFT v7.4.2. server [32]. The alignment was carried out by the FFT-NS-2 progressive method that performs rapid multiple sequence alignment based on a fast Fourier transform (FFT) [33]. This method uses a default scoring matrix derived from Kimura’s two-parameter model [34] (Figure 3). The optimized multiple sequence alignment was further analyzed in MEGA-X V.10.1.7 [35].

We used the annotated complete genome of SARS-CoV-2 isolate Wuhan-Hu-1 as the reference sequence (NC_045512) [13] for the alignment of other complete genome sequences. The flanking regions of the alignment on both ends were truncated to approximately 100 base pairs (bp). Phylogenetic analysis was carried out in the MEGA-X environment using the maximum likelihood estimate of the phylogenetic reconstruction. The phylogeny was tested using a bootstrap-based approach with 100 replications. Evolutionary descendants were inferred using the Tamura–Nei model [36]. The initial tree was generated for the heuristic search by applying the Neighbor-Joining method to a matrix of pairwise distances estimated using the Maximum Composite Likelihood (MCL) approach [37]. The lengths of the branches were measured based on the number of substitutions per site. Codon positions included were 1st, 2nd, 3rd, and Noncoding with the involvement of all 540 genome sequences. This resulted in a total of 30,291 positions in the final dataset.

A nucleotide substitution matrix was generated to determine the probability of specific nucleotide substitutions. Substitution patterns and rates were estimated under the Tamura–Nei model [36]. Transition/transversion bias was estimated using the Maximum Likelihood method, where the substitution pattern and rates were estimated under the Kimura 2-parameter model [38]. Further, to clearly estimate the variation between each pair of genomes among the 540 considered in our analysis, a pairwise distance matrix was generated using the MCL method [39]. The number of base substitutions per site between each sequence was derived as a pairwise distance matrix. Pairwise distances among 540 genomes were visualized by generating a heatmap using gplot:heatmap2 function [40] in the R statistical programming language. The clustering dendrogram was constructed using hierarchical clustering (stat:hclust) [41].

### 2.3. Visualization of Genomic Variations

The complete variations among the 540 genomes were further visualized in Jalview workbench to identify genomic variations such as SNPs and indels [42]. The alignment was applied with a color index based on the percent identity so that identical regions were masked and the nucleotide variations were exposed out of the alignment. This also facilitated visualization of the overall alignment of a large number of genomes in a single window.

### 2.4. Reflection of Genomic Variations in Structural Proteins

Variations arising in the genome subsequently cause changes in the amino acids in the resulting proteins. We focused on amino acid variations in the structural proteins, specifically, surface glycoprotein (S), envelope protein (E), membrane glycoprotein (M), and nucleocapsid (N) proteins, as these proteins are essential targets for developing universal and/or multi-epitope vaccine candidates, as well as potential drug targets [43,44,45,46,47]. The aligned genomic sequences were translated into protein sequences using MEGA-X software and the amino acid variations were identified.

## 3. Results

### 3.1. Phylogenetic Analysis of SARS-CoV-2 Genome Sequences

**Figure 4:**
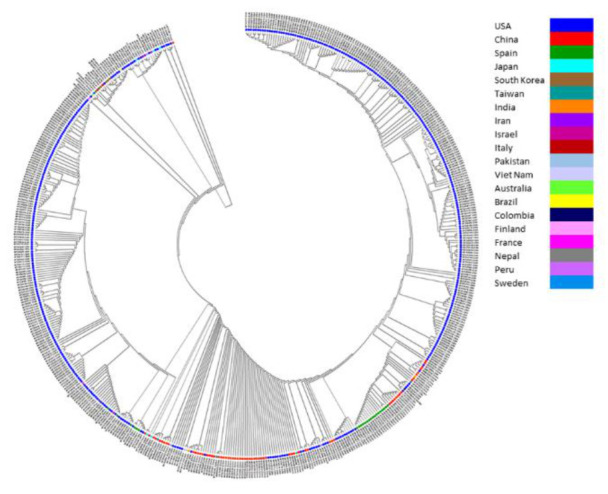
Circular cladogram of 540 genomes of SARS-CoV-2. The cladogram explains the base and spread of SARS-CoV-2 across 20 different countries/territories. The root node was found with an isolate from the Wuhan outbreak, China, and the spread was observed to other countries/territories in the order of USA, Japan, Israel, Pakistan, Iran, Brazil, South Korea, Italy, Spain, Sweden, Australia, Finland, France, Peru, Taiwan territory, Vietnam, India, Nepal, and Colombia.

Initially, a rooted circular cladogram was constructed for 540 SARS-CoV-2 genomes to explain the roots and spread of COVID-19 across the 20 different countries/territories (Figure 4). The root node started with the isolate from China (LR757997) obtained from the Wuhan outbreak. The spread of the nodes started successively, with the isolates from other countries/territories in the following order: USA, Japan, Israel, Pakistan, Iran, Brazil, South Korea, Italy, Spain, Sweden, Australia, Finland, France, Peru, Taiwan, Vietnam, India, Nepal, and Colombia. The order of branching was defined based on the pairwise similarity across the 540 genomes. The nodes at the end of the circular cladogram represent the isolates with an increasing amount of variation in the genomic structure when compared to the reference genome.

**Figure 5:**
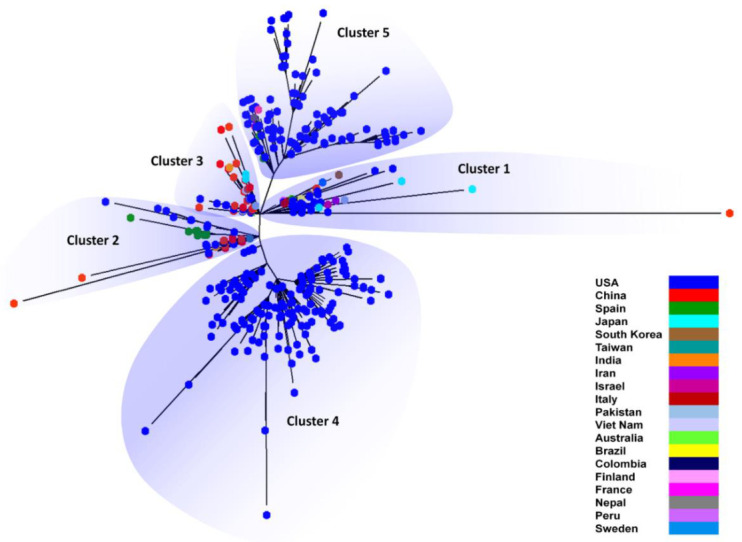
Unrouted phylogram showing the clusters of SARS-CoV-2 isolates across 20 different countries/territories. Cluster 1 represents the root cluster formed with the isolate from the Wuhan outbreak, China.

In order to understand the ancestry and descendants among the 540 isolates, an unrooted phylogenetic tree was constructed (Figure 5). We observed five distinct clusters, among which clusters 4 and 5 were composed nearly entirely of USA isolates and were observed to exhibit variable branch lengths, implying a varying degree of genomic divergence among USA isolates. Interestingly, the most distant nodes were formed by China isolates creating the base rooting nodes, that is, cluster 1 of the tree, and to represent their unique genetic isolation from other nodes of the tree. Several other USA isolates were observed to form distant nodes in clusters 2 and 3, which cover all other countries/territories representing their genetic identity. These nodes would represent the early members or the ancestors in the unrooted tree. The two USA clusters, clusters 4 and 5, were observed to evolve through cluster 2 and cluster 3, respectively, indicating the spread of USA strains from multiple sources. The root bases that indicate the origin of infection were formed by China isolates in clusters 1 (LR757997) and 2 (MT226610, MT123292).

**Figure 6:**
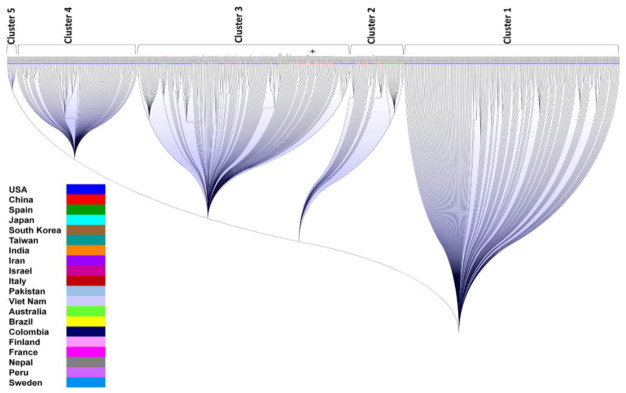
Bootstrap phylogram showing the clusters of SARS-CoV-2 isolates among 20 different countries/territories. A total of 540 genomes of SARS-CoV-2 formed as five distinct clusters. The reference genome from the China outbreak was observed to form in cluster 3 represented with a star. The internal branching depicts the genomic divergence within each cluster.

These phylograms were further subjected to bootstrapping in order to find out the most reliable phylogeny in terms of nucleotide variations. The group of observed genomic variations tended to form clusters. A rooted bootstrap phylogenetic tree was constructed to infer the phylogenetic divergence among the 540 isolates (Figure 6). The bootstrap phylogeny showed five distinct clusters with clear branching points. Clusters 1, 4, and 5 include only USA isolates which are distantly rooted together. This would imply more divergence from one USA cluster to another USA cluster. Cluster 2 contains all Spain isolates and few isolates from China and the USA. This cluster also includes one isolate from India, Columbia, and Taiwan. Cluster 3 is predominantly composed of isolates from China and the USA. The isolates from the other 18 countries/territories were also included in this cluster, along with the reference genome from China. This represents the cluster with internal divergence among the isolates.

We observed many internal branches with variable branch lengths in the USA clusters of both the unrooted and rooted trees, indicating rapid genetic evolution in the SARS-CoV-2 genomes of USA strains.

### 3.2. Multiple Sequence Alignment of SARS-CoV-2 Genome Sequences

**Figure 7:**
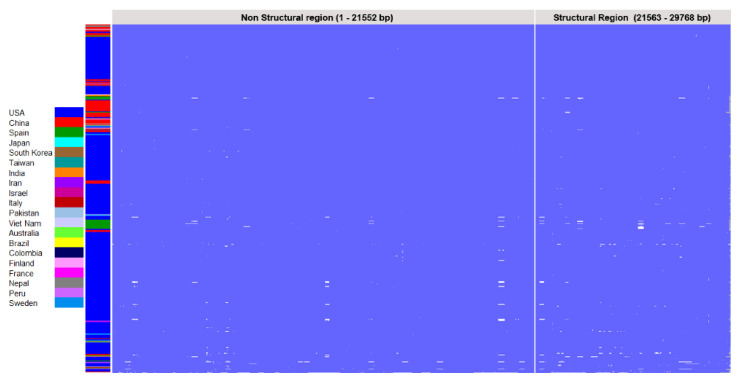
Multiple sequence alignment of 540 SARS-CoV-2 complete genomes. The graphical description of alignment of 540 genomes in a single window from Jalview workbench. The identical regions are masked with blue color and the variations highlighted in white color. The small white bars indicate the deletions in the genomes whereas dots indicate the single nucleotide variations. The 540 genomes are represented countrywise as color bars on the left side of the alignment.

To understand the genomic variations, we performed a multiple sequence alignment for the 540 complete genome sequences. We observed a large number of nucleotide variations throughout the genomic structure, including point mutations and deletions (Figure 7 & File S3). In order to analyze these variations, a nucleotide substitution matrix was generated using the Tamura–Nei method [39]. We found that transitions were far more likely than transversions in these data (approximately 67.58% of substitutions vs. 32.42%, respectively). Nevertheless, given that about a third of the substitutions are transversions, this represents a high tendency towards changes that are more likely to affect the resulting protein [48]. However, this makes the overall transition-to-transversion ratio approximately 2.08. Although the timeframe for accumulating these substitutions is limited, it is interesting that this ratio appears higher than in the SARS coronavirus outbreak in 2003, which had a ratio of approximately 1.1 [49]. However, it is lower than some influenza A viruses [50]. Furthermore, we note that C > T and G > A transitions are more likely than T > C or A > G, which is concordant with the overall nucleotide frequency in these sequences (A: 29.89%, T: 32.12%, C: 18.36%, G: 19.62%). The substitution and Transition/transversion matrices are provided in the supporting information (Files S5 & S6).

**Figure 8:**
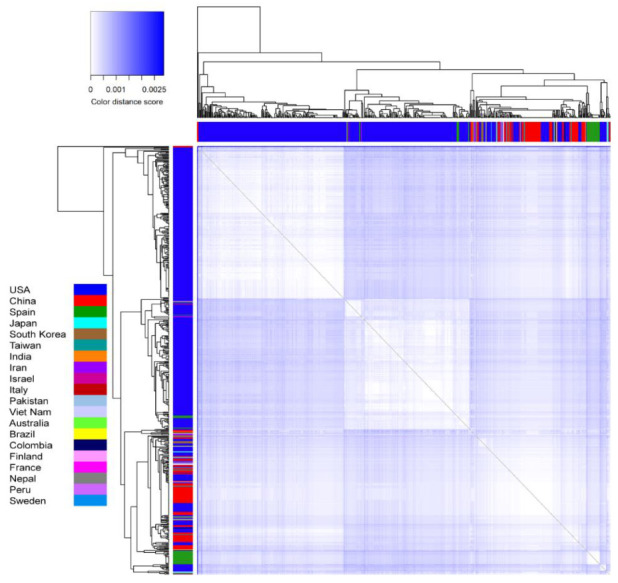
Heat map representing the pairwise distances among 540 genomes. The distance values among each pair of genomes are represented as color gradient. The sequences are represented as color side bars based on the country.

Variations between each pair of genomes were calculated by generating a pairwise distance matrix (File S4). The number of base substitutions per site was calculated for each pair of the 540 genome sequences. All ambiguous positions were removed from each sequence pair (pairwise deletion option). A total of 30,291 positions were identified and the values are represented as a heat map (Figure 8) and the corresponding R-code is provided in the supporting information as File S7. The observations from the distance matrix were consistent with the phylogeny where a large number of genomic variations were observed in the USA strains, representing genomic divergence.

### 3.3. Genomic Variations as Amino Acid Variations in the Structural Proteins

To understand the impact of genomic variations observed in the 540 genomes, we translated surface glycoprotein (S), envelope protein (E), membrane glycoprotein (M), and nucleocapsid (N) structural genes into protein sequences and assessed amino acid variations. Among the 540 genomes, 246 showed amino acid variations in at least one of these four structural genes. The S protein, a major research focus for antigenic determinants, was observed to show a high rate of amino acid variations. Among 540 genomes, 202 showed 34 types of amino acid changes in the S proteins such as L5F, A27V, Y28N, T29I, H49Y, S50L, L54F, N74K, E96D, D111N, F157L, G181V, S221W, T240I, S248R, A344S, A348T, R408I, G476S, V483A, H519Q, A520S, A570V, D614G, H655Y, Q675H, F797C, A930V, D936Y, S940F, A1078V, D1168H, N1178D, and D1259H. The D614G variation was observed in 160 genomes, indicating its conserved nature. The next highest number of amino acid variations was observed in the N-structural protein, where 65 genomes showed 25 types of variations such as D3Y, N4D, P6T, P13L, P14L, S23T, A35T, P46S, D128Y, R185C, S194L, S197L, S202N, R203K, G204R, T205I, A207G, G215S, S232T, G238C, T271I, Q289H, S327L, D343V, and P344S. The maximum frequency of occurrence was found with the R203K variation, observed in 21 genomes, G204R in 19 genomes, and S197L in 15 genomes, indicating the probable conserved nature of these variations in the N-gene. Only three genomes showed two types of amino acid variations such as L37R and P71L in the E protein. Six genomes showed A2S, V70I, and T175M amino acid variations in the M protein (Supplementary File S8).

### 3.4. Mapping of Mutations to the Reported Vaccine Candidates

In order to find out the impact of identified mutations and their implications on vaccine development, we have investigated the reported vaccine candidates from previous studies [51,52,53,54,55,56]. The identified mutations in the structural proteins were mapped with these vaccine candidates and it was found that 23 mutations in S protein, 1 in E, 2 from M, and 7 from N protein were observed to map with the reported B-cell and T-cell epitopes (Supplementary File S9).

## 4. Discussion

In the present study, we observed genomic divergence in SARS-CoV-2 across a relatively short timeframe, based on 540 publicly available and validated genomes collected across 20 different countries/territories. The genomic variations observed in structural genes could serve as useful information for the vaccine development community. The results of this study may also help to address the consequences of genomic diversity in SARS-CoV-2 and its effects on immunogenic response in different patients. The approach we followed to derive the genomic divergence among SARS-CoV-2 genomes establishes several lines of inquiry for the variable immunogenic responses that would be an obstacle for developing a successful vaccine.

The rooted circular cladogram explained the root and spread of SARS-CoV-2 starting from China to 20 different countries/territories. This forms the basis to understand the genesis and dispersion of SARS-CoV-2 infection among 20 different countries/territories, which was defined based on the genomic similarities among 540 genomes. The evolution of ancestors and descendants was further evaluated by the unrooted tree that showed five distinct clusters. The USA clusters were observed to be derived from the clusters of all other countries/territories providing a possible evidence of origin from multiple sources. The root bases formed by China isolates are further demonstrating the root of infection, which was already observed in the circular cladogram. The bootstrapping tree showed similar clustering to that of the unrooted phylogram, indicating the reliability of the clustering process. The varying degrees of branch lengths within the clusters of the bootstrap phylogram indicate high rates of genomic divergence.

Genomic variants, such as indels and substitutions, observed in the multiple sequence alignment of 540 genomes indicate ongoing genetic evolution when compared to the reference genome. The genomic divergence is still continuing as evident from Nextstrain (https://nextstrain.org/sars-cov-2/). Such a high frequency of variations in the genome arising in a short period of time could impact the efficacy of therapeutics and vaccines against COVID-19. There exists a greater need to understand how genomic changes brought about by indels in the genome could impact the antigenicity through protein sequence and structural changes. Hence, these variations may be considered during development of drugs and vaccines against SARS-CoV-2. Identification of critical nucleotide changes reflecting as amino acid changes in the proteins would alter the conformation of the proteins that leads to changes in pathogenicity and antigenicity. Such genetic variations would contribute to diverse antigenic properties resulting in variable immunogenic responses in the patients [57,58]. This would cause an involuntary impact on the rational design of a successful vaccine.

In order to ascertain these variations, we have further evaluated the structure of the SARS-CoV-2 genome and provide interpretations based on our analysis and observations. SARS-CoV-2 contains positive single-stranded RNA genomes with at least six open reading frames. The entire genome is grouped into nonstructural and structural regions (Figure 1). The nonstructural region represents a long ORF1ab that encodes replicase polyproteins required for the replication and transcription of the viral genome. It contains the nonstructural genes nsp1 to nsp16 that encode proteins such as papain-like proteinase (PL); 3-chymotrypsin-like proteinase (3CLPro); RNA-dependent RNA polymerase (RdRp); helicase, 3′-to-5′ exonuclease; endoRNAse; and 2′-O-ribose methyltransferase, which are required for the biochemical and molecular functions of the virus within the host. Hence, the nonstructural genes serve as attractive targets for antiviral drugs [59,60,61]. Further, nsp7 and nsp13 are associated with T-cell immune response [62]. Most of the T-cell epitopes are encoded by structural genes [63]. The structural region encodes four major structural proteins such as S, E, M, and N proteins. These four structural proteins represent ideal targets for the development of universal vaccines as they represent the majority of potential B- and T-cell epitopes [43,44,45,46,47]. These proteins form the protective layer of the virus and are exposed to the host environment at its primary stages of attack. Hence, the induction of immunogenicity in the host primarily depends on these proteins, making them the ideal vaccine candidates. Naturally, variations in the genome can cause changes in amino acids. We have investigated these amino acid changes in the S, E, M, and N proteins (Table S1). The identified mutations were further mapped with the previous vaccine reports where potential vaccine candidates were reported in the structural proteins of SARS-CoV-2 [51,52,53,54,55,56]. A total of 23 mutations in S protein, 1 in E, 2 from M, and 7 from N protein were mapped with the reported vaccine candidates (Table S2). The variations, observed in the proteins, disturb the antigenic determinants and could be responsible for the wide variety of immunogenic responses in each patient. Mutation rate drives viral evolution and genome variability, thereby enabling viruses to escape host immunity and to develop drug resistance. Maria et al. analyzed 220 genomic sequences and stated that the virus is evolving [64]. European, North American, and Asian strains might coexist, each of them characterized by a different mutation pattern. In addition to the mutations in structural proteins, mutations in the RdRp are significant as the virus mutagenic capability depends on the fidelity of RdRp [64]. Over 100 mutations of S protein were studied for their impact on the infectivity and antigenicity by Qianqian et al., and they found that D614G mutation is more infectious [65]. This mutation is also associated with higher viral loads. D614G was also reported to be consistently increasing at regional levels, indicating its fitness advantage [66]. Surprisingly, the same mutation was observed in 160 genomes among 540 in our current study, and also mapped with the vaccine candidates. Another mutation, V483A from S protein, reported to be markedly resistant to monoclonal antibodies (mABs), was observed among six genomes and mapped with the vaccine candidates. These large numbers of amino acid variations in the structural genes suggest that the genomic variations could present challenges in terms of vaccine and treatment development. Such an increasing genomic divergence poses numerous challenges to the research community to fight against COVID-19. Most of the current research on COVID-19 vaccination is based on the identification and characterization of the virulent proteins such as structural proteins of SARS-CoV-2 that interfere with innate and adaptive immune response and are also involved in the interactions with macrophages, T-lymphocytes, and dendritic and epithelial cells. Such immunogenic interactions modulate the host response against viruses to combat pathogenesis [43,67]. The increasing number of mutations causing increase in the genomic divergence would continue to be a challenge in the treatment and vaccine design strategies [58]. The conserved regions of the proteins with no frequency of mutations contribute to stable immunogenicity. Zhang et al. reported that immunodominant (ID) sites of S protein were found to be evolutionarily highly conserved, contributing to potential immunogenicity [68]. Nevertheless, the amino acid variations in SARS-CoV-2 might change the immunogenicity of ID sites, suggesting the careful consideration of epitopes for vaccine design [69].

The findings from the current study highlight the potential impact of genomic variations on protein changes that may stymie the vaccine development process. Information about the possible sites of nucleotide changes and conserved regions of the structural proteins may help other researchers consider specific regions in the proteins that would be avoided as targets for a universal vaccine against SARS-CoV-2 [58,70]. This study also illuminates the important changes for the mechanistic understanding of pathogenicity of SARS-CoV-2 and supports continuing surveillance of mutations to aid with development of a universal vaccine and immunological interventions.

## 5. Conclusions

Currently, most of the world is in the grip of the COVID-19 pandemic, and a vaccine or targeted treatment is urgently needed. Our current study represents an analysis of 540 SARS-CoV-2 complete genomes, collected across 20 different countries/territories. Ongoing genomic divergence was observed among the genomes. In addition, large numbers of nucleotide variations were observed throughout the genome. We analyzed the impact of genomic variations on the structural region of the genome, which is the main target for the development of vaccine candidates. This study suggests these variations be considered in the development of a universal vaccine for COVID-19. We conclude that the continued genomic divergence across successive generations arising due to larger number of nucleotide variations could hinder the development of a universal vaccine. The vaccine research communities could adopt this information to avoid the regions with variations to achieve a successful vaccine against SARS-CoV-2.

## Supplementary Materials

The following are available online at https://www.mdpi.com/2076-393X/8/4/591/s1. File S1. The list of 540 complete genome sequences used in the current study. File S2. The 540 full genome sequences in FASTA format. File S3. The multiple sequence alignment of 540 genomes. File S4. The distance matrix showing the pairwise distances of 540 genomes. File S5. Substitution matrix showing the substitutional probabilities of 540 genomes. File S6. Transition–Transversion matrix showing the transition/transversion bias of 540 genomes. File S7. The R-code generated for the construction of heat map to represent the pairwise distance among 540 genomes. File S8. Genomic variations of SARS-CoV-2 reflected as amino acid changes in the structural proteins. File S9. Mutations mapped onto the reported vaccine candidates.
